# Piericidin A1 Blocks *Yersinia* Ysc Type III Secretion System Needle Assembly

**DOI:** 10.1128/mSphere.00030-17

**Published:** 2017-02-15

**Authors:** Jessica M. Morgan, Miles C. Duncan, Kevin S. Johnson, Andreas Diepold, Hanh Lam, Allison J. Dupzyk, Lexi R. Martin, Weng Ruh Wong, Judith P. Armitage, Roger G. Linington, Victoria Auerbuch

**Affiliations:** a Department of Chemistry and Biochemistry, University of California Santa Cruz, Santa Cruz, California, USA; b Department of Microbiology and Environmental Toxicology, University of California Santa Cruz, Santa Cruz, California, USA; c Department of Biochemistry, University of Oxford, Oxford, United Kingdom; University of Kentucky

**Keywords:** T3SS, *Yersinia*, piericidin, type III secretion system, virulence blocker

## Abstract

The type III secretion system (T3SS) is a bacterial virulence factor expressed by dozens of Gram-negative pathogens but largely absent from commensals. The T3SS is an attractive target for antimicrobial agents that may disarm pathogenic bacteria while leaving commensal populations intact. We previously identified piericidin A1 as an inhibitor of the Ysc T3SS in *Yersinia pseudotuberculosis*. Piericidins were first discovered as inhibitors of complex I of the electron transport chain in mitochondria and some bacteria. However, we found that piericidin A1 did not alter *Yersinia* membrane potential or inhibit flagellar motility powered by the proton motive force, indicating that the piericidin mode of action against *Yersinia* type III secretion is independent of complex I. Instead, piericidin A1 reduced the number of T3SS needle complexes visible by fluorescence microscopy at the bacterial surface, preventing T3SS translocator and effector protein secretion. Furthermore, piericidin A1 decreased the abundance of higher-order YscF needle subunit complexes, suggesting that piericidin A1 blocks YscF needle assembly. While expression of T3SS components in *Yersinia* are positively regulated by active type III secretion, the block in secretion by piericidin A1 was not accompanied by a decrease in T3SS gene expression, indicating that piericidin A1 may target a T3SS regulatory circuit. However, piericidin A1 still inhibited effector protein secretion in the absence of the T3SS regulator YopK, YopD, or YopN. Surprisingly, while piericidin A1 also inhibited the *Y. enterocolitica* Ysc T3SS, it did not inhibit the SPI-1 family Ysa T3SS in *Y. enterocolitica* or the Ysc family T3SS in *Pseudomonas aeruginosa*. Together, these data indicate that piericidin A1 specifically inhibits *Yersinia* Ysc T3SS needle assembly.

**IMPORTANCE** The bacterial type III secretion system (T3SS) is widely used by both human and animal pathogens to cause disease yet remains incompletely understood. Deciphering how some natural products, such as the microbial metabolite piericidin, inhibit type III secretion can provide important insight into how the T3SS functions or is regulated. Taking this approach, we investigated the ability of piericidin to block T3SS function in several human pathogens. Surprisingly, piericidin selectively inhibited the Ysc family T3SS in enteropathogenic *Yersinia* but did not affect the function of a different T3SS within the same species. Furthermore, piericidin specifically blocked the formation of T3SS needles on the bacterial surface without altering the localization of several other T3SS components or regulation of T3SS gene expression. These data show that piericidin targets a mechanism important for needle assembly that is unique to the *Yersinia* Ysc T3SS.

## Abstract (precis)

The bacterial type III secretion system (T3SS) is widely used by both human and animal pathogens to cause disease yet remains incompletely understood. Deciphering how some natural products, such as the microbial metabolite piericidin, inhibit type III secretion can provide important insight into how the T3SS functions or is regulated. Taking this approach, we investigated the ability of piericidin to block T3SS function in several human pathogens. Surprisingly, piericidin selectively inhibited the Ysc family T3SS in enteropathogenic *Yersinia* but did not affect the function of a different T3SS within the same species. Furthermore, piericidin specifically blocked the formation of T3SS needles on the bacterial surface without altering the localization of several other T3SS components or regulation of T3SS gene expression. These data show that piericidin targets a mechanism important for needle assembly that is unique to the *Yersinia* Ysc T3SS.

## INTRODUCTION

Piericidins are a family of natural products produced by *Actinobacteria* that resemble coenzyme Q, also known as ubiquinone, a molecule heavily involved in electron transport in prokaryotes and eukaryotes alike (1). The piericidin family member piericidin A1 was originally shown to be a potent inhibitor of NADH oxidase in purified beef heart mitochondria (2). Subsequently, analysis of piericidin A1-resistant mutants of the bacterium *Rhodobacter capsulatus* suggested the molecular target to be the 49-kDa subunit of NADH-ubiquinone oxidoreductase, referred to as complex I (3). Complex I is a large protein complex containing at least 40 subunits with a mass of ~1 MDa and is a central component of the electron transport chain in eukaryotes and prokaryotes (4). This complex functions to translocate protons and is integral in generating a proton gradient for ATP synthesis and other processes such as powering protein secretion. Piericidin A1 acts by blocking the reduction of coenzyme Q by complex I (3, 5). Many bacteria are insensitive to piericidin, likely because of differences in complex I subunit composition and/or substrate and cofactor binding interfaces (6) that possibly prevent piericidins from binding. Piericidin A1 has been explored as a potential anticancer agent, as treatment with nanomolar concentrations of the compound prevented upregulation of the glucose receptor GRP78, resulting in rapid cell death in glucose-starved HT-29 cells (7), a human cultured colon cancer cell line. More recently, Kang et al. described piericidin A1 as an inhibitor of quorum sensing in *Erwinia carotovora*, a potato plant pathogen, although the mechanism underlying this remains unclear (8).

Recently, we identified piericidin A1 and the piericidin derivative Mer-A-2026B as inhibitors of the bacterial type III secretion system (T3SS) (9). The T3SS is a complex nanomachine composed of more than 20 different structural proteins that assemble into a needle-like injection apparatus that punctures eukaryotic cells and acts as a conduit through which bacterial effector proteins enter the host cell cytosol (10). Protein secretion through this apparatus requires a proton motive force (11), as well as the ATPase YscN (12), and the apparatus is assembled in three stages. The basal body, assembled first, is composed of rings that are embedded in the inner and outer membrane of the bacterium and bridge the gap of the periplasmic space. Upon completion of the basal body, secretion of the “early” T3SS substrates begins. The SctF needle subunit, referred to as YscF in *Yersinia*, is secreted at this stage and polymerizes from the basal body to form a hollow needle protruding ~60 nm into the extracellular space (13). Upon completion of needle assembly, a substrate specificity switch leads to secretion of the “middle” substrates, which comprise the pore or translocon complex (10). These three additional proteins, in *Yersinia*, called YopD, YopB, and LcrV, are secreted and form the translocon complex at the tip of the newly polymerized YscF needle (14). YopK (YopQ in *Yersinia enterocolitica*) has been shown to regulate the translocation of the pore-forming translocon proteins YopB and YopD, as well as effector Yops (15). In addition to its role as a translocon protein, YopD is thought to form a complex with LcrQ and LcrH to bind Yop mRNA and the 30S ribosomal subunit, inhibiting translation until the T3SS is actively secreting cargo, including LcrQ and YopD (16, 17). The final substrate switch, to the secretion of “late” cargo, T3SS effector proteins called Yops in *Yersinia* and regulatory proteins, is triggered *in vivo* by host cell contact, which may be sensed by the translocon (18). In *Yersinia*, this response can be mimicked through the chelation of calcium (19, 20). Therefore, secretion of late Yops is prevented in the presence of high calcium concentrations and permitted at low calcium concentrations. In *Yersinia*, the regulatory proteins YopN and TyeA prevent the secretion of Yops prior to host cell contact or a decrease in the calcium concentration (21, 22).

We previously identified piericidin A1 as an inhibitor of the T3SS in *Yersinia pseudotuberculosis*. In this study, we characterized the activity of piericidin A1 on the T3SSs of the related gut pathogens *Y. pseudotuberculosis* and *Y. enterocolitica*, as well as the opportunistic pathogen *Pseudomonas aeruginosa*.

## RESULTS

### Complex I inhibition by piericidin does not underlie its ability to block type III secretion.

Piericidin A1 inhibits complex I of the electron transport chain in at least some bacterial species (3). Type III secretion requires a proton motive force to translocate substrates; treatment with the proton uncoupler carbonyl cyanide *m*-chlorophenylhydrazone (CCCP) abolishes secretion (see Fig. S1A in the supplemental material). Therefore, it is possible that piericidins block type III secretion by blocking proton motive force generation. To investigate this possibility, we tested the effects of pyridaben and rotenone, two structurally distinct compounds with complex I inhibitory activity in mitochondria and certain bacteria, on Yop secretion in *Y. pseudotuberculosis*. Pyridaben and rotenone did not significantly block secretion of the T3SS effector YopE, while piericidin inhibited YopE secretion by 50% (see Fig. S1B), suggesting that compounds with complex I inhibitory activity do not all inhibit type III secretion.

10.1128/mSphere.00030-17.1 FIG S1  (A) *Y. pseudotuberculosis* type III secretion was determined after the addition of CCCP, a proton decoupler, to test the role of proton motive force in type III secretion. (B) *Y. pseudotuberculosis* type III secretion of YopE was measured in the presence of piericidin A1 and the complex I inhibitors rotenone and pyridaben. Shown are the average results of five independent experiments ± the standard error of the mean. *, *P* < 0.03 by analysis of variance with Tukey’s honestly significant difference *post hoc* test on all four samples. Download FIG S1, TIF file, 0.6 MB. Copyright © 2017 Morgan et al. 2017 Morgan et al. This content is distributed under the terms of the Creative Commons Attribution 4.0 International license.

**FIG 1:**
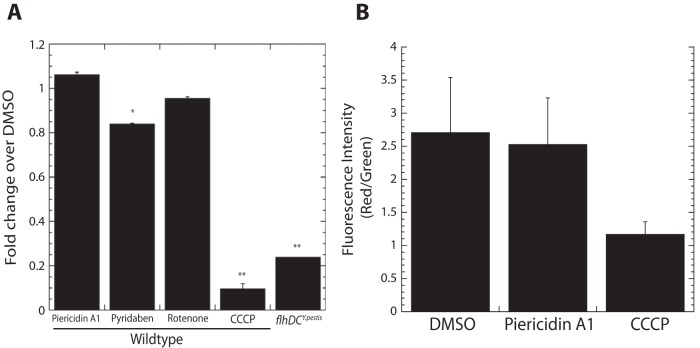
Complex I inhibition activity of piericidin A1 does not underlie its T3SS inhibitory activity. (A) Motility was analyzed by spotting 1-µl aliquots of WT *Y. pseudotuberculosis* or a nonmotile mutant onto motility medium in the presence of DMSO or piericidin A1 (71 µM). Shown are the average results of four independent experiments ± the standard error of the mean. *, *P* < 0.001 by analysis of variance with Bonferroni’s *post hoc* test. (B) The proton motive force of *Y. pseudotuberculosis* was measured in the presence of DMSO or piericidin A1 (71 µM) with JC-1 dye. The protonophore CCCP was added as a negative control. The ratio of red/green fluorescence intensity indicates membrane potential. Shown are the average results of two independent experiments.

As both nonflagellar and flagellar T3SSs require proton motive force, we tested whether piericidin A1 affects *Y. pseudotuberculosis* motility in a soft agar migration assay. Piericidin A1 (71 µM) did not inhibit *Yersinia* motility, while the proton gradient uncoupler CCCP did (Fig. 1A). In addition, piericidin A1 did not alter the speed or direction of *Yersinia* motility, as observed by video microscopy (data not shown). Finally, we evaluated any effect piericidin A1 may have on membrane potential directly by using the membrane potential indicator dye JC-1 and observed no significant change (Fig. 1B). These data indicate that piericidin A1 does not inhibit the flagellar T3SS and that piericidins do not prevent the generation of a proton motive force needed to support type III secretion in *Yersinia*. Furthermore, these results suggest that piericidin A1 does not target a structure common to the flagellar and nonflagellar Ysc T3SSs or a regulator common to the two secretion systems, such as Hfq (23).

Coenzyme Q shows some structural similarity to piericidins and associates with complex I during electron transport (24) (see Fig. S2). Coenzyme Q is also sold as a dietary supplement for human consumption (25). Therefore, we tested the ability of coenzyme Q to inhibit type III secretion in *Yersinia*. Unlike piericidin, coenzyme Q did not inhibit YopE secretion in *Y. pseudotuberculosis* (see Fig. S3).

10.1128/mSphere.00030-17.2 FIG S2  Structures of piericidin A1 and coenzyme Q. Download FIG S2, TIF file, 0.5 MB. Copyright © 2017 Morgan et al. 2017 Morgan et al. This content is distributed under the terms of the Creative Commons Attribution 4.0 International license.

10.1128/mSphere.00030-17.3 FIG S3  Coenzyme Q does not inhibit the Ysc T3SS. Yop secretion from *Y. pseudotuberculosis* grown under T3SS-inducing conditions in the presence of coenzyme Q, piericidin A1, or DMSO (control) was analyzed. Each concentration of coenzyme Q used is shown with the corresponding equal-volume DMSO control. At higher DMSO percentages, bacterial fitness is compromised, leading to decreased secretion, as seen with the largest DMSO volume used. The image shown is representative of three biological replicates. Download FIG S3, TIF file, 1.7 MB. Copyright © 2017 Morgan et al. 2017 Morgan et al. This content is distributed under the terms of the Creative Commons Attribution 4.0 International license.

### Piericidin A1 inhibits the *Y. pseudotuberculosis* and *Y. enterocolitica* Ysc T3SS but not the *Y. enterocolitica* Ysa T3SS.

**FIG 2:**
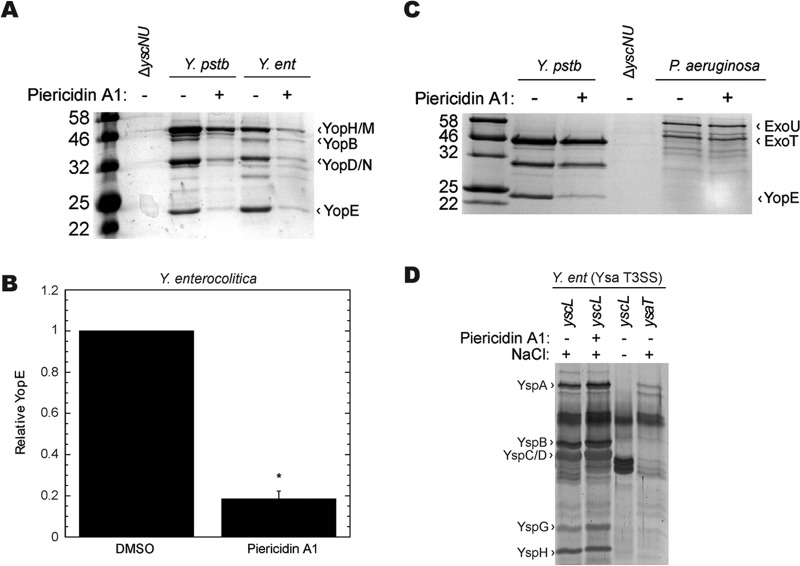
Piericidin A1 blocks the Ysc T3SS in enteropathogenic *Yersinia* but does not block the Ysc family T3SS in *P. aeruginosa* or the Ysa T3SS in *Y. enterocolitica*. The relative efficiency of effector protein secretion into the culture supernatant was analyzed following bacterial growth under T3SS-inducing conditions in the presence or absence of piericidin A1 (71 µM). The secretome was precipitated with TCA, separated by SDS-PAGE, and visualized by staining with Coomassie blue (A to C) or SYPRO Ruby (D). (A to C) Ysc family T3SS: *Y. pseudotuberculosis* (*Y*. *pstb*), *Y. enterocolitica* (Y. *ent*), and *P. aeruginosa* were grown in low-calcium medium at 37°C. The *Y. pseudotuberculosis* Δ*yscNU* mutant strain was used as a negative control for Yop secretion, as it does not express a Ysc T3SS (50). *, *P* < 0.0001 by Student’s *t* test. (D) Ysa T3SS. *Y. enterocolitica* was grown under high-salt conditions at 26°C. The values to the left of the blots in panels A and C are molecular sizes in kilodaltons.

As we did not observe inhibition of the flagellar T3SS by piericidin A1, we tested its ability to inhibit the activity of T3SSs more closely related to the *Y. pseudotuberculosis* Ysc T3SS. Piericidin A1 reduced YopE secretion in *Y. enterocolitica* by 80% through the Ysc family T3SS in that organism (Fig. 2A and B), similar to its previously reported effect on YopE secretion in *Y. pseudotuberculosis* (Fig. 2A) (9). However, piericidin A1 did not block ExoU or ExoT secretion in *P. aeruginosa* (Fig. 2C), suggesting that piericidin does not inhibit the *Pseudomonas* T3SS, which is closely related to the Ysc T3SS (26). *P. aeruginosa* is known to exclude many antibiotics (27). Therefore, if the piericidin target of action is intracellular, it is possible that lack of target accessibility explains why piericidin cannot inhibit the *P. aeruginosa* T3SS.

To test the ability of piericidin to block a T3SS outside the Ysc family in an organism where the target is known to be accessible, we took advantage of the fact that *Y. enterocolitica* encodes a second T3SS on its chromosome, called the Ysa system (28, 29). The Ysa T3SS is induced under high-salt conditions at low temperatures (26°C), reflecting its possible role during early stages of infection (28). We used a *Y. enterocolitica yscL* mutant defective in the Ysc T3SS to focus on the Ysa T3SS. We found that piericidin did not affect levels of the secreted Ysa effector proteins called Ysps (Fig. 2D). As a control, the *yscL* mutant did not show Ysp secretion under low-salt conditions. Lastly, a *ysaT* mutant previously shown to be defective in Ysa secretion (30) did not secrete Ysps. These data show that while piericidin can inhibit the Ysc T3SS in *Y. enterocolitica*, this compound cannot block the Ysa T3SS in the same organism.

### Piericidin A1 traps T3SS effector proteins inside bacterial cells independently of the YopN, YopD, and YopK T3SS regulatory proteins.

**FIG 3:**
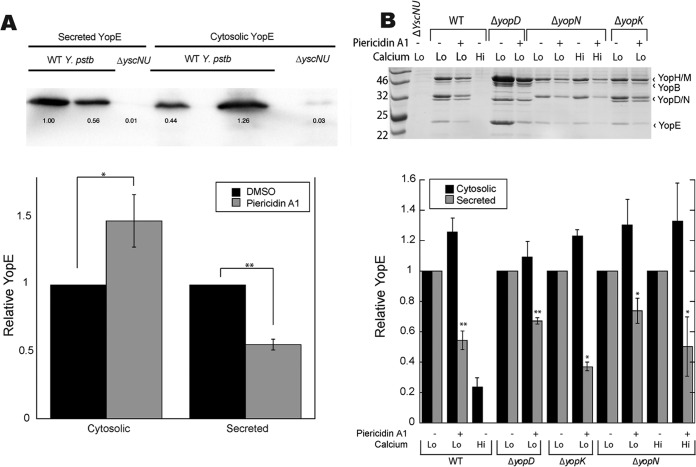
Piericidin A1 traps YopE in the bacterial cytoplasm. (A) Cytosolic and secreted YopE of WT *Y. pseudotuberculosis* treated with DMSO or piericidin A1 (71 µM) was detected by immunoblot assay. Shown are the average results of eight or nine independent experiments ± the standard error of the mean. *, *P* < 0.03; **, *P* < 0.0001 (Student’s *t* test). (B) Representative SDS-PAGE analysis showing the secretome of WT and T3SS regulatory mutant strains of *Y. pseudotuberculosis* treated with piericidin A1 (71 µM) under high- and low-calcium conditions (top). Quantitation of cytosolic and secreted YopE for the same strains and conditions (bottom) in four independent experiments ± the standard error of the mean. *, *P* < 0.05; **, *P* ≤ 0.0005 (Student’s *t* test). The values to the left of the blot in panel B are molecular sizes in kilodaltons.

To begin to understand how piericidin inhibits *Yersinia* type III secretion, we tested whether piericidin A1 blocks type III secretion by blocking the expression of T3SS structural components or effectors. Transcript levels of the T3SS ATPase YscN and the effector YopE were unchanged following piericidin A1 treatment (see Fig. S4), suggesting that piericidin A1 prevents T3SS activity through a posttranscriptional mechanism. Therefore, we tested whether piericidin A1 could prevent YopE protein expression by analyzing levels of secreted and intracellular YopE. While the pool of secreted YopE decreased upon piericidin treatment, intracellular YopE levels were significantly higher (Fig. 3A), indicating that piericidin A1 blocks the secretion, not the translation, of Yops. To determine if piericidin A1 blocks Yop secretion by targeting a T3SS regulatory component, we tested the ability of piericidin A1 to inhibit Yop secretion in *Y. pseudotuberculosis* mutants lacking YopD, YopK, or YopN. In all cases, piericidin A1 was able to inhibit Yop secretion (Fig. 3B) and YopE buildup in the bacterial cytosol remained unaffected (Fig. 3B; see Fig. S5). This indicates that piericidin inhibits the T3SS independently of the YopD, YopK, and YopN regulators.

10.1128/mSphere.00030-17.4 FIG S4  Piericidin A1 does not affect T3SS gene transcript levels. *Y. pseudotuberculosis* was grown in M9 medium at 37°C, and transcript levels were measured by real-time PCR as described in Materials and Methods. The average value ± the standard error of the mean of three independent experiments is shown. 16S rRNA levels were used as the reference. Download FIG S4, TIF file, 0.1 MB. Copyright © 2017 Morgan et al. 2017 Morgan et al. This content is distributed under the terms of the Creative Commons Attribution 4.0 International license.

10.1128/mSphere.00030-17.5 FIG S5  Piericidin A1 leads to accumulation of Yops in the bacterial cytosol of WT *Y. pseudotuberculosis*, as well as a *Yersinia* mutant lacking the regulatory protein YopD, YopK, or YopN. These are additional biological replicates of the experiments shown in Fig. 3B. Download FIG S5, TIF file, 0.1 MB. Copyright © 2017 Morgan et al. 2017 Morgan et al. This content is distributed under the terms of the Creative Commons Attribution 4.0 International license.

### Piericidin A1 decreases the number of T3SS needles associated with the bacterial surface but does not alter the localization of basal body components.

**FIG 4:**
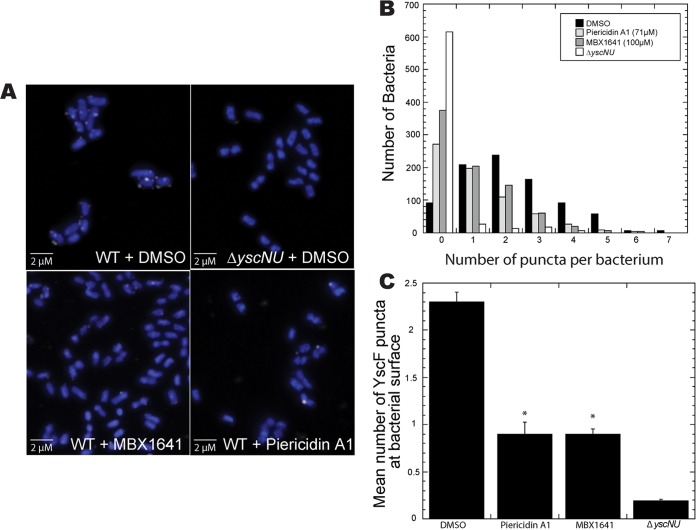
Treatment of *Yersinia* bacteria with piericidin A1 leads to a decrease in bacterium-associated YscF puncta. (A) Representative confocal microscopy images of *Y. pseudotuberculosis* stained for T3SS puncta following treatment with DMSO or the T3SS inhibitors MBX-1641 and piericidin A1 (71 µM). (B) Histogram showing the number of T3SS puncta per bacterium following treatment with DMSO or T3SS inhibitors. (C) Average number of T3SS puncta per bacterium following drug treatment in four independent experiments ± the standard error of the mean. *, *P* < 0.0001 by analysis of variance with Bonferroni’s *post hoc* test.

To determine if piericidin A1 inhibits Yop secretion by blocking the assembly of the T3SS needle, we incubated *Y. pseudotuberculosis* in the presence or absence of piericidin A1 and measured the number of T3SS needle complexes by staining the bacteria with antibodies recognizing the needle subunit YscF. One antibody, raised against full-length, purified YscF, was previously used to analyze YscF puncta in *Y. pseudotuberculosis* (31), while the other antibody was raised against a YscF peptide for this study (see Materials and Methods). Regardless of which antibody was used, piericidin A1 reduced the number of bacterium-associated YscF puncta by approximately half (Fig. 4 and data not shown), suggesting that piericidin inhibits Yop secretion by blocking the formation of T3SS complexes, blocking the secretion of needle subunits, and/or leading to the assembly of nonfunctional needles that are not recognized by anti-YscF antibodies. We compared this phenotype to that of an inhibitor whose molecular target has been suggested through suppressor mutation analysis to be the YscF homolog in *P. aeruginosa*, PscF (32). Piericidin A1 reduced YscF puncta by the same magnitude as the PscF phenoxyacetamide inhibitor MBX1641 (Fig. 4).

To determine whether the reduction in needle numbers was caused (or accompanied) by a corresponding reduction in basal body assembly, we analyzed the subcellular localization of three sorting platform proteins, YscK, YscL, and YscQ, and of the basal body ring protein YscD by monitoring enhanced green fluorescent protein (EGFP)-linked alleles of these proteins in *Y. enterocolitica* (33). While we observed a slight increase in spot intensity upon piericidin A1 treatment compared to that of the dimethyl sulfoxide (DMSO) control, there was no statistically significant change in the number of spots per unit of cell length (see Fig. S6 and S7), suggesting that the main effect of piericidin is not on the level of localization of the cytosolic or basal body T3SS components. Instead, piericidin A1 may block the secretion of T3SS cargo, including YscF or YscF needle assembly.

10.1128/mSphere.00030-17.6 FIG S6  Piericidin A1 does not affect the localization of cytosolic components of the T3SS apparatus. *Y. enterocolitica* strains expressing EGFP-YscQ, EGFP-YscK, EGFP-YscD, or mCherry-YscL were grown under T3SS-inducing conditions in the presence of 71 μM piericidin A1 or DMSO, and fluorescent foci were quantified by using MatLab as previously described (45). The intensity of spots and the number of spots per pixel are plotted for each protein. Shown are the data from two independent replicates. The Mann-Whitney nonparametric test was used to calculate *P* values. Download FIG S6, TIF file, 1.6 MB. Copyright © 2017 Morgan et al. 2017 Morgan et al. This content is distributed under the terms of the Creative Commons Attribution 4.0 International license.

10.1128/mSphere.00030-17.7 FIG S7  Representative images of foci imaged for each strain shown in Fig. S6. Download FIG S7, TIF file, 2.5 MB. Copyright © 2017 Morgan et al. 2017 Morgan et al. This content is distributed under the terms of the Creative Commons Attribution 4.0 International license.

### Piericidin A1 blocks YscF higher-order structure formation and decreases the secretion of middle and late T3SS substrates.

**FIG 5:**
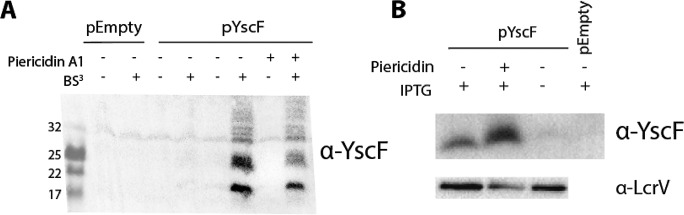
Treatment of *Yersinia* bacteria with piericidin A1 affects YscF needle polymerization. (A) Cross-linking of YscF polymers on *Y. pseudotuberculosis* in the presence or absence of piericidin A1 (71 µM). The image shown is representative of five independent replicates. (B) Secreted YscF and LcrV were detected by immunoblot assay after TCA precipitation of supernatants from *Y. pseudotuberculosis* treated with DMSO or piericidin A1 (71 µM).

To determine if piericidin affects YscF needle polymerization, we employed a BS^3^ cross-linking assay (34). We detected a decrease in higher-order YscF polymers between the control and piericidin-treated bacteria (Fig. 5A), suggesting that piericidin A1 decreases YscF needle assembly. In further support of this conclusion, we observed an accumulation of monomeric YscF in the supernatant upon piericidin A1 treatment (Fig. 5B). Taken together, these data support a model in which piericidin A1 prevents the proper assembly of YscF polymers, resulting in an accumulation of monomeric YscF in the supernatant.

The T3SS basal body secretes its cargo in a hierarchical manner, with early (ex-YscF), middle (ex-LcrV), and late (ex-YopE) substrates (35). As piericidin A1 caused an increase in monomeric YscF, decreased polymeric YscF, and decreased YopE in the supernatant, we tested the effect of piericidin A1 on the secretion of LcrV and YopD. As seen for late substrates, secretion of LcrV and YopD was decreased upon piericidin A1 treatment (Fig. 5B and data not shown), suggesting that piericidin blocks YscF needle assembly, thereby inhibiting the secretion of middle and late T3SS substrates.

### Piericidin A1 inhibition of type III secretion is reversible, and this reversibility is independent of new protein synthesis.

**FIG 6:**
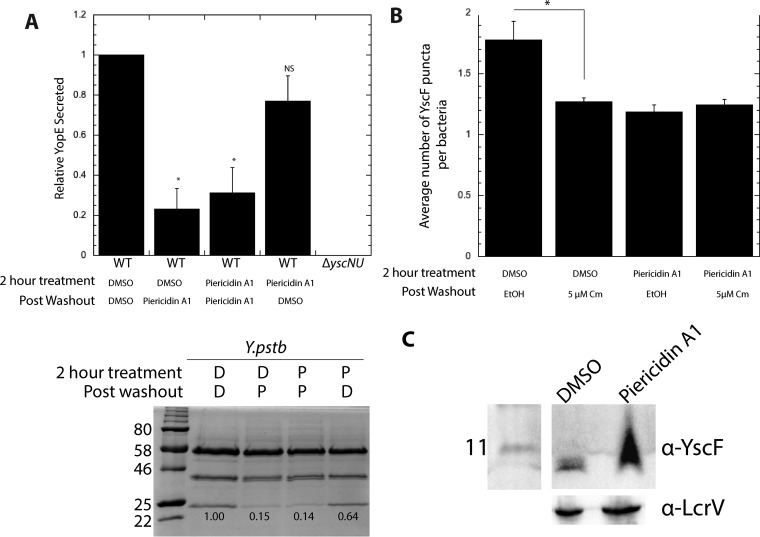
The action of piericidin A1 on YopE secretion is independent of new protein synthesis. (A) Relative levels of secreted YopE from *Y. pseudotuberculosis* incubated with piericidin A1 (71 µM) or DMSO for an initial 2 h, followed by a washout and resuspension in fresh low-calcium medium and either DMSO (D) or piericidin A1 (P; 71 µM) for another 45 min of incubation. Shown are the average results of four independent experiments ± the standard error of the mean (top) and a representative gel (bottom). The values to the left of the gel are molecular sizes in kilodaltons. *, *P* < 0.002 by analysis of variance with Tukey’s honestly significant difference *post hoc* test on all four samples. (B) Average number of YscF puncta following treatment with piericidin A1 (71 µM) and/or the translation inhibitor chloramphenicol. Shown are the average results of four independent experiments ± the standard error of the mean. *, *P* < 0.02 by Student’s *t* test. (C) Cytosolic YscF was detected by immunoblot assay for *Y. pseudotuberculosis* pYscF treated with DMSO or piericidin A1 (71 µM). YscF expression was induced with 10 µM IPTG. The lanes containing the ladder and samples shown were separated by unrelated samples in the intervening lanes on the same blot and are therefore shown divided by a white border.

We next sought to determine whether piericidin inhibition of Yop secretion is reversible. We incubated *Y. pseudotuberculosis* in the presence of piericidin A1 for 2 h under T3SS-inducing conditions, washed the bacteria to remove the compound, and allowed them to secrete T3SS effectors in fresh low-calcium medium with or without piericidin A1 for an additional 45 min. As expected, incubation of *Y. pseudotuberculosis* with piericidin A1 for the duration of the experiment significantly reduced the secretion of YopE (Fig. 6A). It should be noted that cells that were exposed to piericidin A1 only during the last 45 min of the experiment still had a significant decrease in secretion, indicating that piericidin can inhibit type III secretion after T3SS expression has already been induced. However, if piericidin was removed for the final 45 min of incubation, the YopE secretion level approached that of control bacteria treated with DMSO for the entire experiment (Fig. 6A). These data indicate that the action of piericidins on the T3SS is reversible, either because the piericidin A1 block in YscF needle assembly is reversible or because new needle complexes are assembled during the piericidin-free 45 min of incubation. To address the latter possibility, we repeated the washout treatment in the presence or absence of the translation inhibitor chloramphenicol during the final 45 min of incubation after the removal of piericidin A1 and analyzed YscF puncta. While chloramphenicol significantly decreased the number of YscF puncta associated with bacteria not exposed to piericidin A1, chloramphenicol did not alter the number of YscF puncta on *Yersinia* bacteria pretreated with piericidin A1 (Fig. 6B). These data suggest that reversal of piericidin A1 inhibition by washing away of the compound does not depend on new protein synthesis; rather, it allows for previously constructed needle complexes to regain secretion activity once piericidin is removed or for new needles to be polymerized from preformed needle subunits. Indeed, we observed an accumulation of cytoplasmic YscF in piericidin-treated bacteria under T3SS-inducing conditions (Fig. 6D), indicating that while YscF can be secreted in the presence of piericidin A1, the block in polymerization may eventually lead to a backup of YscF in the cytosol. This cytoplasmic pool may then be secreted and polymerized into a functional T3SS needle once piericidin is removed.

## DISCUSSION

In this study, we characterize the activity of piericidin A1 as an inhibitor of the bacterial T3SS in *Y. pseudotuberculosis* and *Y. enterocolitica*. Piericidin A1 was originally described as an inhibitor of complex I in the mitochondrial electron transport chain (2) and in some prokaryotes (3). However, the data presented here demonstrate that piericidin A1 inhibits the T3SS independently of complex I. In addition, piericidin A1 was shown to affect quorum-sensing pathways in *E. carotovora* (8). However, studies on quorum sensing in *Yersinia pestis* indicate that quorum sensing negatively regulates the expression of T3SS genes (36). As our study shows that piericidin A1 does not affect T3SS gene expression, piericidin activity on the *Yersinia* T3SS is predicted to be independent of any effect on quorum sensing. Instead, piericidin blocks the assembly of the *Yersinia* YscF needle and subsequently the secretion of middle and late T3SS substrates without altering the localization of T3SS basal body components or inhibiting the secretion of early substrates. Furthermore, the effect of piericidin is specific, as it does not influence the *Yersinia* Ysa or flagellar T3SS. Thus, we propose that piericidins have an alternative molecular target specific to the Ysc T3SS in pathogenic *Yersinia* involved in YscF needle assembly.

Our data suggest that the molecular target of piericidin is unique to the Ysc secretion system and common to the two enteropathogenic *Yersinia* species but is either absent from or not well enough conserved in *P. aeruginosa*, which expresses a Ysc family T3SS. Alternatively, if piericidin must cross the bacterial cell envelope to carry out its T3SS inhibitory activity, *P. aeruginosa* may exclude or efflux piericidins. *P. aeruginosa* is known to have a high degree of drug resistance because of its unusually restrictive outer membrane permeability (27). However, we can conclude that piericidins do not inhibit the more distantly related *Y. enterocolitica* Ysa T3SS, though piericidin can inhibit the Ysc T3SS in this species.

Piericidin treatment reduced the number of YscF needle puncta visible by fluorescence microscopy at the *Yersinia* surface. Each punctum may represent multiple T3SS needles located in proximity to one another (37), so our quantitation of approximately two puncta per bacterium likely underestimates the actual number of T3SS needles. Nevertheless, both piericidin and the needle subunit-targeting T3SS inhibitor MBX-1641 reduced the number of YscF puncta to fewer than one per bacterium. This suggests that both compounds either disrupt needle assembly or alter needle conformation such that needles cannot be readily recognized by our antibody. As we observed a similar piericidin-induced decrease in YscF puncta when a different antibody raised against full-length YscF was used (data not shown), the latter explanation is unlikely. Our data showing that piericidin decreases the abundance of higher-order YscF polymers visible upon chemical cross-linking provides evidence for the former possibility.

Interestingly, while piericidin A1 treatment attenuates the secretion of middle and late T3SS substrates, it does not decrease effector protein expression but instead causes an accumulation of effectors in the bacterial cytosol. These results are surprising given the known T3SS feedback loop; Yop mRNA levels are positively regulated by active secretion (38). YopD and LcrQ have been shown to prevent Yop translation prior to host cell contact, and their subsequent secretion out of the bacterial cytosol through the T3SS relieves this repression (39). YopN is a regulatory protein involved in the triggering of effector secretion following host cell contact. Thus, we hypothesized that piericidin may block a regulatory pathway controlled by host cell contact, effectively causing a host contact “blind” phenotype. However, piericidin blocked type III secretion in the absence of YopD and YopN just as in wild-type (WT) bacteria. In addition, piericidin also inhibited type III secretion in the absence of YopK, a regulatory protein that controls T3SS translocon-mediated pore formation, a process recently linked to sensing of host cell contact. Collectively, these data suggest that piericidin may block the ability of the YscF T3SS needle to mediate Yop secretion by altering YscF needle assembly in a reversible manner that does not impact the T3SS feedback loop.

Whether piericidins have potential as antimicrobial therapeutics remains unclear. While their specificity and efficacy on preformed T3SSs are attractive, they have two obvious limitations. First, the micromolar concentration required to observe T3SS inhibition is impractical for use in the clinic as a treatment or in prophylaxis. A second, and related, concern is the compound’s toxicity to mammalian cells. Several groups have found piericidins to be poorly tolerated in animal models, likely because of their inhibition of murine mitochondrial complex I (40). A solution may lie in structure-activity relationship studies. Perhaps more promising is the use of these molecules as laboratory tools. Given the surprising result that piericidin blocks the secretion of middle and late substrates without impacting the T3SS feedback loop, the compounds might be used to better understand T3SS regulatory pathways, as well as YscF needle formation and function. Furthermore, the compounds could be used to explore host-pathogen interactions involving temporary T3SS inactivation, as type III secretion can be restored by washing away the compound. Lastly, understanding why piericidin specifically targets the *Yersinia* Ysc T3SS may reveal novel mechanisms by which *Yersinia* bacteria have evolved to control the activity of this major virulence factor.

## MATERIALS AND METHODS

### Bacterial strains and growth conditions.

The bacterial strains used in this study are listed in Table 1. *Y. pseudotuberculosis* was grown in 2×YT (yeast extract-tryptone) at 26°C with shaking overnight. The cultures were back diluted into low-calcium medium (2×YT plus 20 mM sodium oxalate and 20 mM MgCl_2_) to an optical density at 600 nm (OD_600_) of 0.2 and grown for 1.5 h at 26°C with shaking, followed by 1.5 h at 37°C to induce Yop synthesis as previously described (41). *P. aeruginosa* was grown in Luria-Bertani broth at 37°C with shaking. *Y. enterocolitica* was grown in 1% tryptone–0.5% yeast extract at 26°C with shaking overnight.

**TABLE 1:** Bacterial strains used in this study

Strain	Mutation	Reference(s)
*Y. pseudotuberculosis*		
IP 2666pIB1	None (WT)	49
Δ*yscNU* mutant	Δ*yscNU*	50
Δ*yopN* mutant	Δ*yopN*	34
Δ*yopK* mutant	Δ*yopK*	This study
Δ*yopD* mutant	Δ*yopD*	This study
*flhDC*^*Y. pestis*^ mutant	Δ*yopHEMOJ flhDC*^*Y. pestis*^	41
pYscF mutant	Δ*yopHEMOJ ΔYscF*/pTCR99-YscF	34
pEmpty mutant	Δ*yopHEMOJ ΔYscF*/pTCR99 empty vector	34
*P. aeruginosa* PA103^a^	None (WT)	51, 52
*Y. enterocolitica*		
*yscL* mutant	*yscL*::mini-Tn*5* KM2	53
*ysaT* mutant	*ysaT*::Tn*Mod*-RKm'	54

a Expresses ExoU and ExoT effectors.

The full-length *yopD* deletion mutation (42), leaving only the first three and last three amino acids of YopD, was constructed with the primers described in reference 43. The *yopK* deletion mutation was introduced into *Y. pseudotuberculosis* with a pSR47s-derived suicide plasmid obtained from Molly Bergman (42). *Y. pseudotuberculosis* Δ*yopD* and Δ*yopK* mutants were isolated as previously described (42).

### Piericidin A1.

Piericidin A1 was isolated from *Actinomyces* sp. strain RL09-253-HVS-A, which was isolated from a marine sediment sample collected by scuba at Point Estero, CA, at a depth of 50 feet. This *Actinomyces* strain was identified as a *Streptomyces* sp. based on the typical morphology of streptomycetes, which form dry powdery spores on agar plates, and by sequencing of the 16S rRNA gene (data not shown). From a large-scale culture (4 liters) of the strain in SYP medium, we obtained an organic extract that was subject to a stepwise eluotropic gradient on a 10-g C_18_ Sep Pak cartridge (methanol [MeOH]-H_2_O gradient, 40 ml of 10%, 20% [fraction A], 40% [fraction B], 60% [fraction C], 80% [fraction D], and 100% [fraction E] MeOH and then 100% ethyl acetate [fraction F]). A 0.33-g sample of fraction D was obtained, and the active constituent was purified by C_18_ reverse-phase high-performance liquid chromatography (gradient profile, 58% to 88% MeOH–0.02% formic acid–H_2_O, 2 ml/min [Synergi 10 Fusion-RP column; Phenomenex, USA]; retention time, 30.5 min) to give 2.7 mg of piericidin A1. Electrospray ionization-time of flight high-resolution mass spectrometry analysis confirmed the molecular formula C_25_H_37_NO_4_. This structure was confirmed by one-dimensional ^1^H nuclear magnetic resonance spectroscopy on a Varian Unity Inova spectrometer at 600 MHz and comparison with values in the literature. Piericidin A1 is commercially available; however, mass spectroscopy of commercially available piericidin A1 found it to be impure (data not shown). We have observed that these impurities can lead to inconsistent and artifactual results (data not shown). The concentration of piericidin A1 used in our experiments, 71 µM, is approximately twice the 50% inhibitory concentration for Yop translocation (9).

### Anti-YscF antibody.

The anti-YscF antibody used in this study was raised in rabbits against the *Y. pseudotuberculosis* YscF peptide KDKPDNPALLADLQH (Pacific Immunology).

### Measurement of membrane potential.

Bacteria were grown under the type III secretion-inducing conditions described above, and membrane potential was measured as previously described (44). Briefly, during the last 30 min of the 37°C incubation, JC-1, a fluorescent dye widely used to measure membrane potential, was added. As a control, the protonophore CCCP was used. Samples were imaged with an LSM 5 PASCAL laser scanning microscope (Zeiss) fitted with a Plan-Apochromat 63×/1.4 oil differential inference contrast (DIC) objective and analyzed with the LSM 510 software (Zeiss). Quantification of image intensities was performed with ImageJ.

### Type III secretion assay.

Visualization of *Y. pseudotuberculosis* T3SS cargo secreted in broth culture was performed as previously described (41). *Y. pseudotuberculosis* low-calcium medium cultures were grown for 1.5 h at 26°C. We added compounds or DMSO and switched the cultures to 37°C for another 2 h. Coenzyme Q (Sigma) was resuspended in warm DMSO. For the type III secretion reversibility assay, following the 2-h 37°C step, cultures were washed and incubated with piericidin A1 (71 µM) or the equivalent volume of DMSO for another 45 min before secreted proteins were isolated (45). To test dependence on protein synthesis, following the 2-h 37°C step, cultures were washed and incubated with 5 µM chloramphenicol or an equal volume of 100% ethanol for another 45 min before secreted proteins were isolated or cells were fixed for microscopy experiments. To test the effect of CCCP, a compound that causes uncoupling of the proton gradient, on secretion, overnight cultures were diluted 1:40 in brain heart infusion (BHI) broth and grown for 2 h at 37°C. CCCP was then added at 10 µM, and the culture was shaken for 5 min. Secretion was then induced by the addition of 5 mM EGTA and 10 mM MgCl_2_. Cultures were collected at 5, 30, and 60 min after T3SS induction.

Overnight cultures of *P. aeruginosa* were washed and resuspended in LB supplemented with 5 mM EGTA and 20 mM MgCl_2_ with piericidin A1 (71 µM) or DMSO and grown at 37°C for 3 h (46). For *Y. enterocolitica* Ysa secretion, overnight cultures were grown in 1% tryptone–0.5% yeast extract (no-salt L medium) at 26°C with shaking overnight. The cultures were back diluted into L medium with either no salt or 290 mM NaCl added and incubated for 6 h at 26°C with shaking as previously described (30). Cultures were centrifuged at 13,200 rpm for 10 min at room temperature. Supernatants were transferred to a new Eppendorf tube. For *Y. enterocolitica* supernatants, the upper two-thirds only was removed and centrifuged again. The upper two-thirds was removed once again and passed through a 0.22-µm filter prior to trichloroacetic acid (TCA) precipitation. Ten percent (final concentration) TCA was added, and the mixture was vortexed vigorously. Samples were incubated on ice for 20 min and then centrifuged at 13,200 rpm for 15 min at 4°C. The pellet was resuspended in final sample buffer (FSB) plus 20% dithiothreitol (DTT). Samples were boiled for 15 min prior to being subjected to 12.5% SDS-PAGE. Sample loading was normalized for the bacterial density (OD_600_) of each sample. *Y. pseudotuberculosis* and *P. aeruginosa* samples were visualized by Coomassie blue staining, while *Y. enterocolitica* Ysa samples were visualized with SYPRO Ruby (Molecular Probes). Densitometric quantification of the bands was done with Image Lab software (Bio-Rad) with the relevant DMSO-treated *Y. pseudotuberculosis* YopE band set to 1.00.

### Secreted and cytosolic YopE Western blot analysis.

Supernatant samples were obtained as described above for the type III secretion assay. In parallel, after the supernatant was collected, the pellet was resuspended in the same volume of FSB plus 20% DTT as the corresponding supernatant sample. The pellet samples were then boiled for 15 min. Cytosolic and secreted protein samples were subjected to 12.5% SDS-PAGE and transferred to a blotting membrane (Immobilon-P) with a trans-Blot semidry transfer cell (Bio-Rad). Blots were blocked overnight in Tris-buffered saline with Tween 20 and 5% skim milk and probed with a YopE primary antibody and a horseradish peroxidase-conjugated secondary antibody (Santa Cruz Biotech). Following visualization, densitometric quantification of the bands was performed with Image Lab software (Bio-Rad) with the first DMSO-treated WT *Y. pseudotuberculosis* YopE band set to 1.00.

### Detection of secreted monomeric YscF.

As we could not detect secreted monomeric YscF from WT bacteria, presumably because it becomes rapidly polymerized into the needle structure, we used a strain of *Y. pseudotuberculosis* that ectopically expresses YscF from an isopropyl-β-d-thiogalactopyranoside (IPTG)-inducible pTCR99A plasmid (pYscF). Cultures were grown overnight in 2×YT with 100 µg/ml carbenicillin. The following day, cultures were back diluted to an OD_600_ of 0.2 in low-calcium medium plus carbenicillin and grown at 26°C for 1.5 h. At the 37°C shift, 10 µM IPTG and either 71 µM piericidin A1 or DMSO were added to the cultures, which were incubated for 2 h. After incubation, bacteria were normalized by OD_600_ and TCA precipitation was preformed as described above.

### Cross-linking assay.

*Yersinia* bacteria carrying YscF on an IPTG-inducible plasmid (pYscF) were grown under the T3SS-inducing conditions described above. At the 37°C shift, 10 µM IPTG was added to induce YscF expression. After a 2-h induction, the cells were collected and the crosslinker BS^3^ was added as previously described (34). Samples were boiled in Laemmli buffer and pulled through a 25-gauge needle several times to shear genomic DNA. Samples were loaded onto a 16.5% Tris-Tricine gel (Bio-Rad) and run at 120 V. Proteins were transferred to a polyvinylidene difluoride membrane (Immobilon-P) with a Mini-Trans-Blot cell (Bio-Rad) and probed with a YscF antibody (1:10,000) overnight, followed by a horseradish peroxidase-conjugated secondary antibody (Santa Cruz Biotech).

### Motility assay.

Following overnight growth, bacterial cultures were diluted to an OD of 2.5. One microliter of WT *Y. pseudotuberculosis* or a nonmotile mutant expressing *flhDC*^*Y. pestis*^ (41) was spotted onto motility medium (1% tryptone, 0.25% agar) in six-well plates. Each well contained either 0.3% DMSO or 71 µM piericidin A1, rotenone, or pyridaben. The plates were stored at 26°C for 24 h before the diameter of swimming motility from the center was measured. To increase bacterial visibility, 24 mM 3-(4,5-dimethyl-2-thiazolyl)-2,5-diphenyl-2H-tetrazolium bromide was added on top of the motility agar just before imaging.

### Real-time PCR.

Overnight bacterial cultures grown in M9 were back diluted and grown in M9 at 37°C to induce type III secretion as described in reference 44, in the presence of DMSO, piericidin A1, rotenone, or pyridaben. RNA was isolated with an RNeasy Plus Micro kit (Qiagen) according to the manufacturer’s instructions. SYBR green PCR master mix (Applied Biosystems) was used for quantitative PCRs (qPCRs) according to the manufacturer’s instructions at a 60°C annealing temperature. The primers used are listed in Table S1. The YopE primers were designed with primer 3 software (http://fokker.wi.mit.edu/primer3/input.htm). Results were analyzed with the Bio-Rad CFX software.

10.1128/mSphere.00030-17.8 TABLE S1  qPCR primers used in this study. Download TABLE S1, DOCX file, 0.05 MB. Copyright © 2017 Morgan et al. 2017 Morgan et al. This content is distributed under the terms of the Creative Commons Attribution 4.0 International license.

### Immunofluorescence staining of YscF.

Visualization of YscF was carried out as previously described (31), with the following modifications. Coverslips were blocked overnight with phosphate-buffered saline with Tween 20 (PBST) plus 3% bovine serum albumin (PBST-BSA) at 4°C. The blocking solution was removed, an anti-YscF primary antibody was added at 1:10,000 in PBST-BSA, and the mixture was rocked at 4°C for 4 h. The coverslips were carefully rinsed in ice-cold PBST plus 0.1% Tween 20 several times, incubated with an Alexa Fluor 594-conjugated anti-rabbit secondary antibody (Invitrogen) at 1:10,000 in PBST-BSA, rocked at 4°C for 3 h, and rinsed again in ice-cold PBST plus 0.1% Tween 20 several times. The coverslips were then stained for nuclear material with Hoechst 33342 (Thermo Scientific) at 1:10,000 in PBST-BSA and left in the dark at room temperature for 30 min. The coverslips were washed in ice-cold PBST plus 0.1% Tween 20 several times, allowed to dry briefly, mounted onto glass coverslips with ProLong Gold (Thermo Scientific), and sealed with clear nail polish. Images were taken with a Zeiss Axio Imager Z2 widefield microscope under 63×/1.4 oil immersion with Zen software and pseudocolored and merged in FIJI. The conditions were blinded, and the puncta associated with a single bacterium were counted.

### YscK, YscL, YscD, and YscQ analysis.

*Y. enterocolitica* bacteria expressing N-terminal fluorescent fusions of cytosolic T3SS components, EGFP-YscK, mCherry-YscL, EGFP-YscQ, or EGFP-YscD joined by a glycine-rich flexible 13-amino-acid linker and expressed from the native genetic environment on the virulence plasmid (47, 48) were grown overnight in BHI broth containing nalidixic acid (35 μg/ml) and diaminopimelic acid (80 μg/ml). Main cultures were inoculated to an OD of 0.12 in the same medium additionally complemented with 5 mM EDTA, 10 mM MgCl_2_, and 0.4% glycerol. Cultures were grown for 1.5 h at 28°C. A 71 μM concentration of piericidin A1 or the equivalent volume of DMSO was added, and the cultures were switched to 37°C for another 3 h. A 1.5-μl volume of the culture was layered on a patch of 1.5% agarose in M22 medium supplemented with 80 μg/ml diaminopimelic acid, 5 mM EDTA, 10 mM MgCl_2_, 0.4% glycerol, and either 71 μM piericidin A1 or the equivalent volume of DMSO. Fluorescence and DIC images of z stacks (*n* = 4, Δz = 250 nm) were taken on a Deltavision optical sectioning microscope (Applied Precision) equipped with a UPlanSApo 100×/1.35 oil objective (Olympus). DIC frames were imaged for 10 ms, and mCherry and EGFP frames were imaged for 1 s with appropriate filter sets. Detection and quantification of fluorescent foci were performed as described in reference 33.

### Statistical analysis.

Statistical analysis was performed with Kaleidagraph (Synergy Software) or GraphPad as specified in each figure legend.
