# Supplementary figures and images for: Piericidin A1 Blocks Yersinia Ysc Type III Secretion System Needle Assembly
Source: mSphere. 2017 Feb 15;2(1):e00030-17. doi: 10.1128/mSphere.00030-17 (PMC5311113; doi:10.1128/mSphere.00030-17)

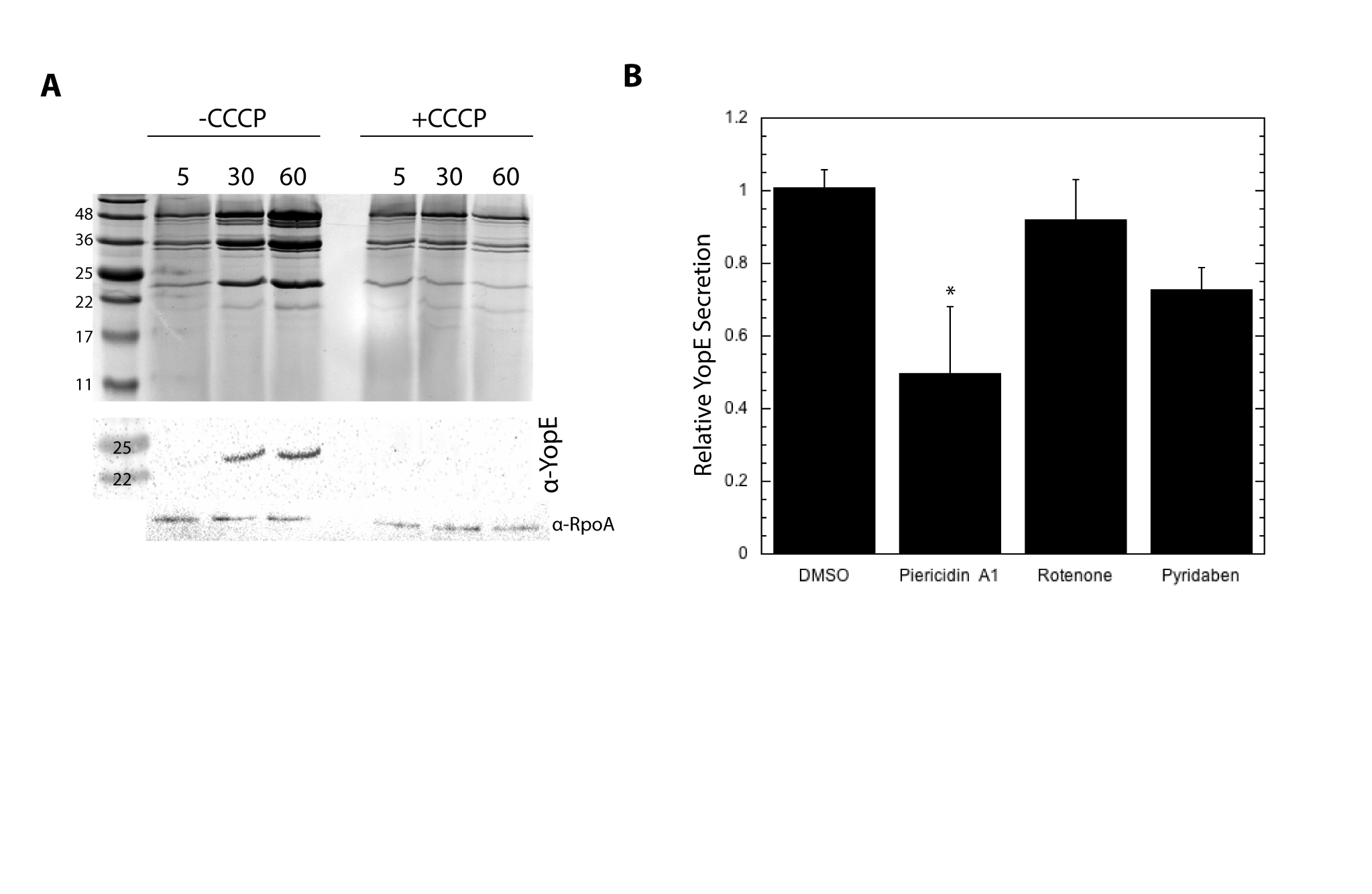

Supplement: FIG S1 [file sph001172233sf1.tif]

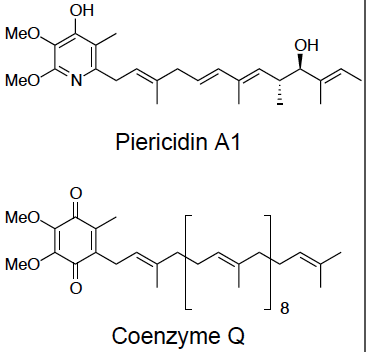

Supplement: FIG S2 [file sph001172233sf2.tif]

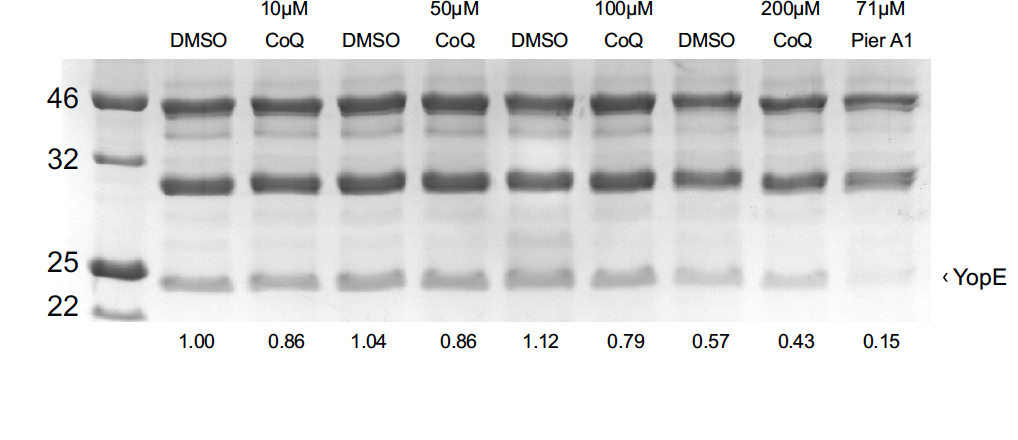

Supplement: FIG S3 [file sph001172233sf3.tif]

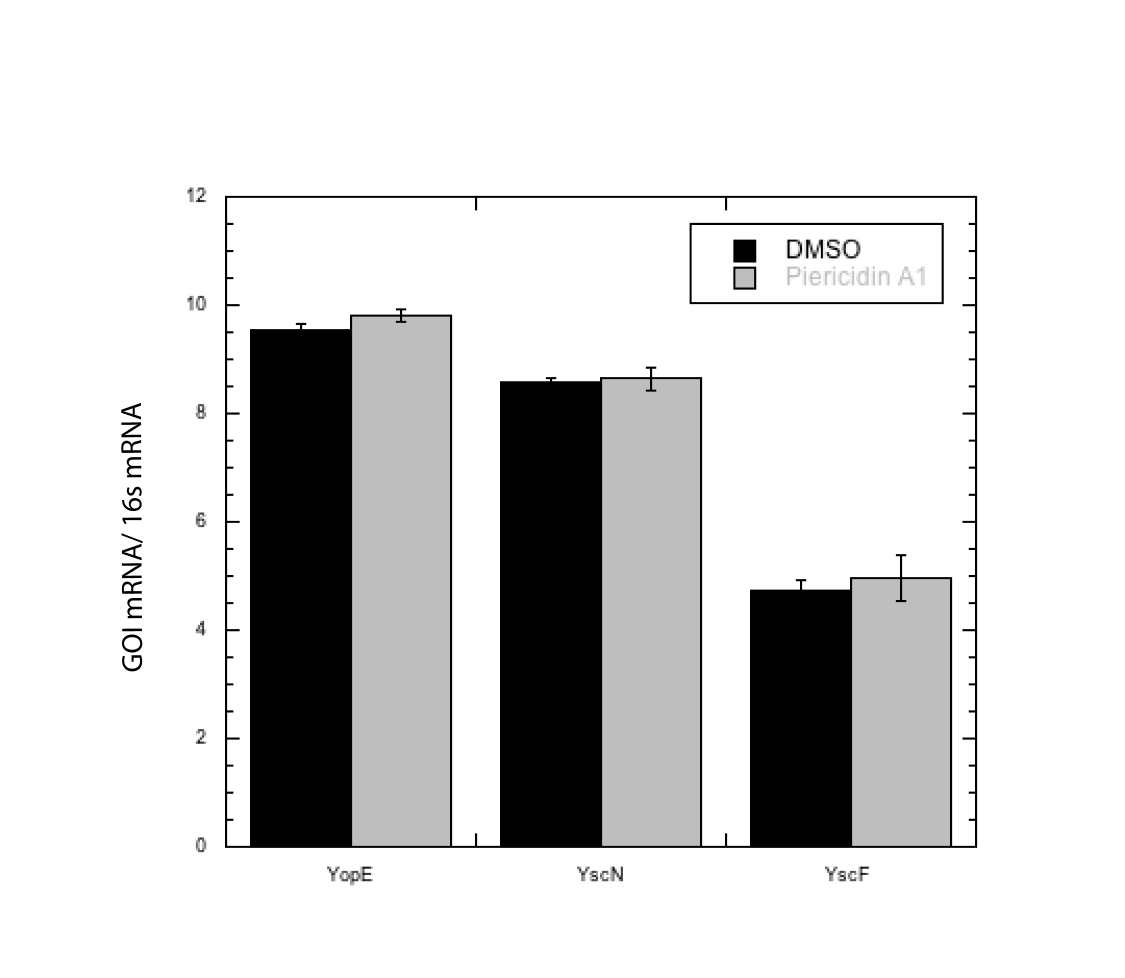

Supplement: FIG S4 [file sph001172233sf4.tif]

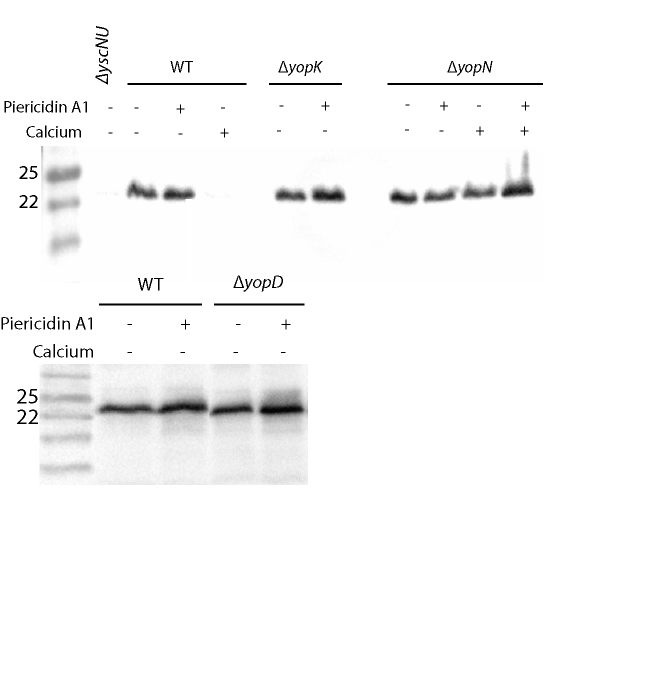

Supplement: FIG S5 [file sph001172233sf5.tif]

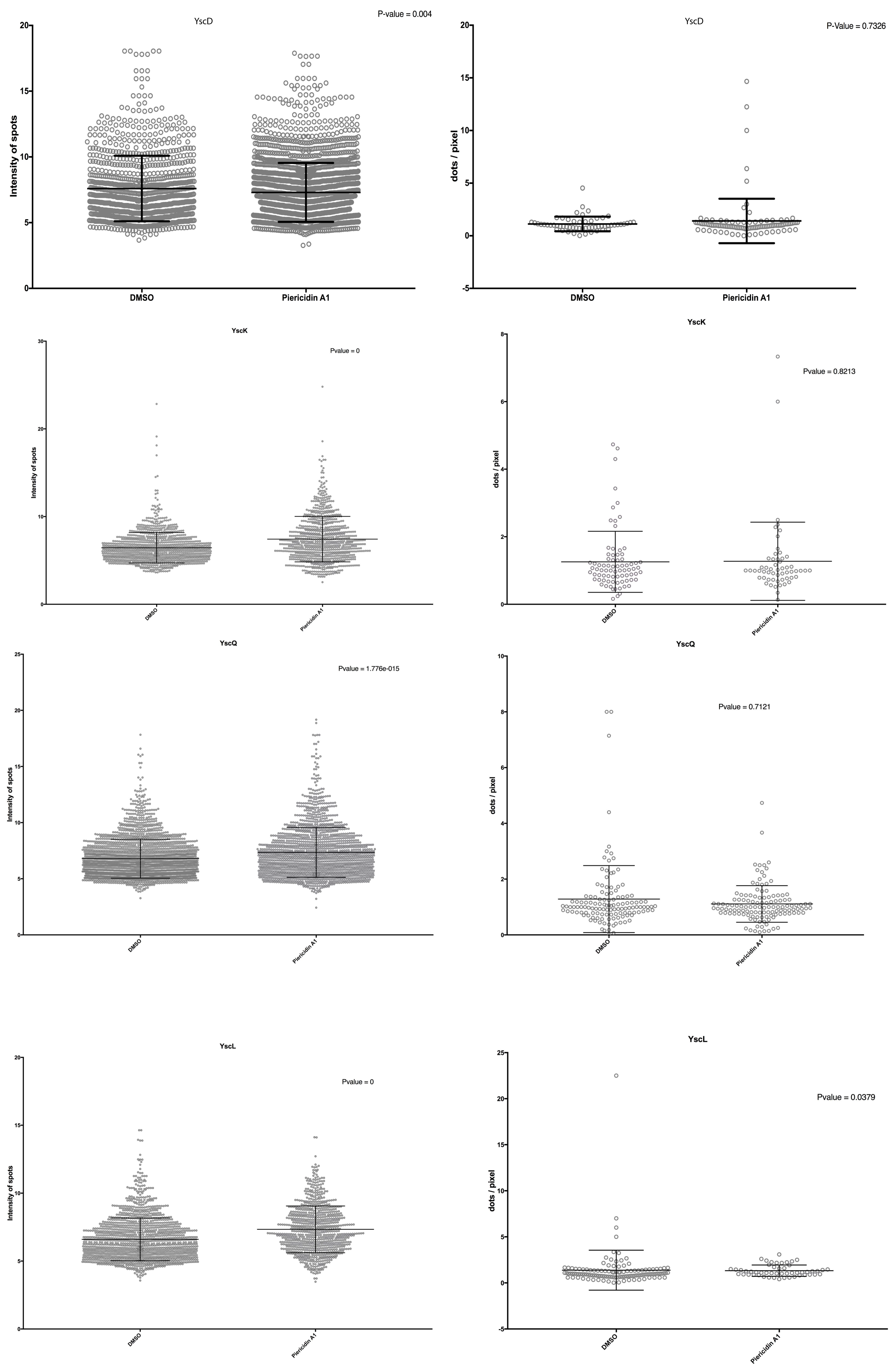

Supplement: FIG S6 [file sph001172233sf6.tif]

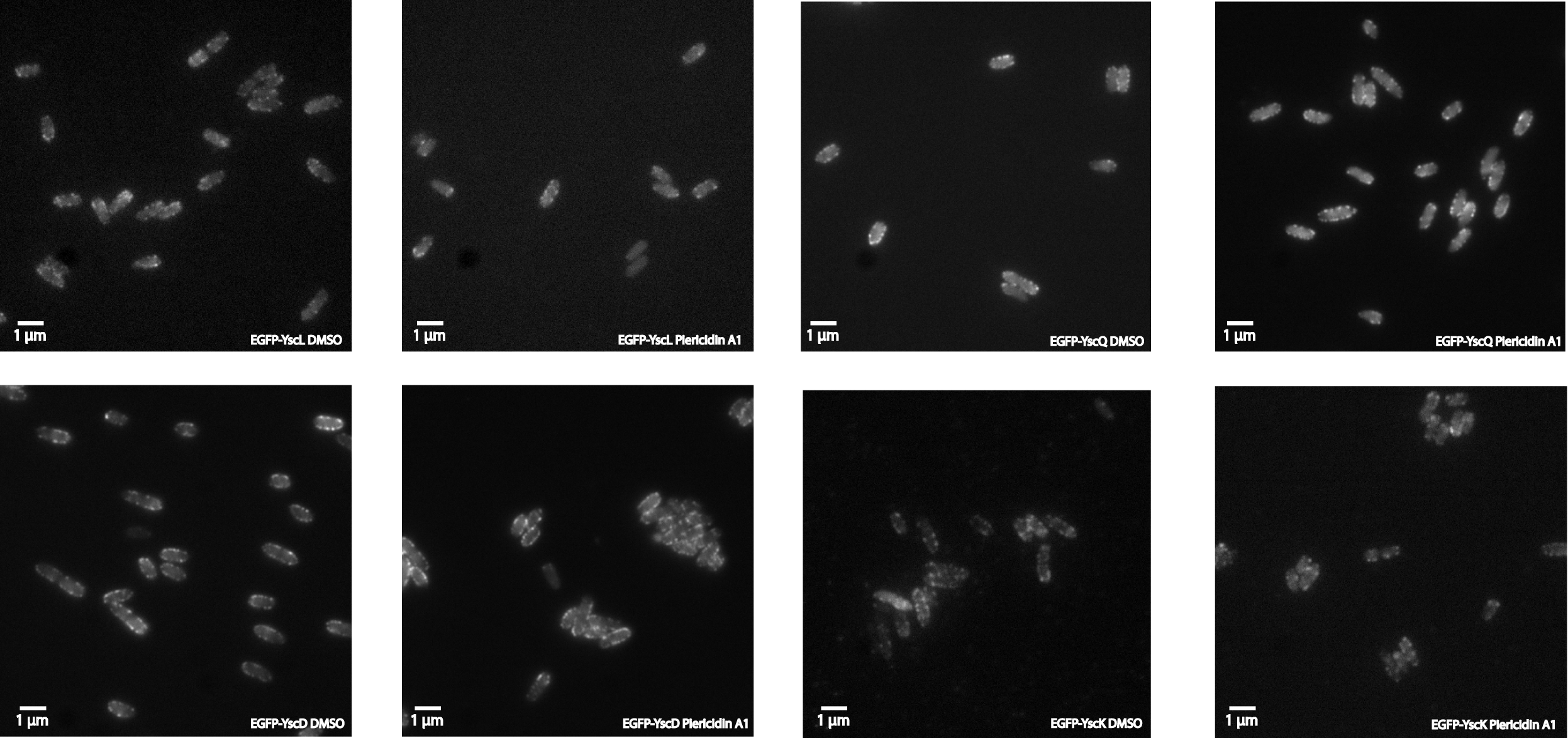

Supplement: FIG S7 [file sph001172233sf7.tif]
