# Supplementary material for: Piericidin A1 Blocks Yersinia Ysc Type III Secretion System Needle Assembly
Source: mSphere. 2017 Feb 15;2(1):e00030-17. doi: 10.1128/mSphere.00030-17 (PMC5311113; doi:10.1128/mSphere.00030-17)
Supplement: TABLE S1 [file sph001172233st8.docx]

**Supplementary Table 1. Quantitative PCR primers used in this study.**

| Name | Sequence | Reference |
| --- | --- | --- |
| Fq*yopE* | CCATAAACCGGTGGTGAC | This study |
| Rq*yopE* | CTTGGCATTGAGTGATACTG | This study |
| Fq*yscN* | CTTCGCTTATTCGTAGTGCT | (1) |
| Rq*yscN* | TCGCCTAAATCAGACTCAAT | (1) |
| Fq*yscF* | TCTCTGGATTTACGAAAGGA | (1) |
| Rq*yscF* | GCTTATCTTTCAATGCTGCT | (1) |
| Fq16s | AGCCAGCGGACCACATAAAG | (2) |
| Rq16s | AGTTGCAGACTCCAATCCGG | (2) |

1) Miller HK, Kwuan L, Schwiesow L, Bernick DL, Mettert E, Ramirez HA, Ragle JM, Chan PP, Kiley PJ, Lowe TM, Auerbuch V. 2014. IscR is essential for *Yersinia pseudotuberculosis* type III secretion and virulence. PLoS Pathog 10:e1004194. <https://doi.org/10.1371/journal.ppat.1004194>.

2) Merriam JJ, Mathur R, Maxfield-Boumil R, Isberg RR. 1997. Analysis of the *Legionella pneumophila fliI* gene: intracellular growth of a defined mutant defective for flagellum biosynthesis. Infect Immun 65:2497–2501.
